# Sonographic Assessment of Uterine Biometry for the Diagnosis of Diffuse Adenomyosis in a Tertiary Outpatient Clinic

**DOI:** 10.3390/jpm12101572

**Published:** 2022-09-23

**Authors:** Diego Raimondo, Lucia Lazzeri, Antonio Raffone, Matteo Giorgi, Benedetta Orsini, Ludovica Verrelli, Jacopo Lenzi, Antonio Travaglino, Lucia De Meis, Antonio Mollo, Errico Zupi, Renato Seracchioli, Paolo Casadio

**Affiliations:** 1 Division of Gynecology and Human Reproduction Physiopathology, IRCCS Azienda Ospedaliero-Universitaria di Bologna, 40138 Bologna, Italy; 2 Department of Molecular and Developmental Medicine, Obstetrics and Gynecological Clinic, University of Siena, 53100 Siena, Italy; 3 Department of Medical and Surgical Sciences, University of Bologna, 40138 Bologna, Italy; 4 Department of Biomedical and Neuromotor Sciences, Alma Mater Studiorum University of Bologna, 40138 Bologna, Italy; 5 Gynecopathology and Breast Pathology Unit, Department of Woman and Child’s Health Sciences and Public Health, Fondazione Policlinico Universitario A. Gemelli IRCCS, 00168 Rome, Italy; 6 Department of Advanced Biomedical Sciences, University of Naples “Federico II”, 80138 Naples, Italy; 7 Gynecology and Obstetrics Unit, Department of Medicine, Surgery and Dentistry “Schola Medica Salernitana”, University of Salerno, 84084 Baronissi, Italy

**Keywords:** adenomyosis, biometry, diagnosis, globular uterus, ultrasonography, ultrasound

## Abstract

Background: to compare several uterine biometric parameters at transvaginal ultrasound (TVUS) between adenomyosis and non-adenomyosis uteri and evaluate their role for the diagnosis of diffuse adenomyosis. Methods: prospective observational study conducted between the 1 February 2022 and the 30 April 2022. In this case, 56 patients with TVUS diagnosis of adenomyosis were included. A 1:1 ratio age and parity-matched group of non-adenomyosis patients was selected. We compared sonographic uterine biometric parameters (longitudinal (LD), anteroposterior (APD) and transverse (TD) diameters, volume, simple and complex diameter ratios) and investigated their diagnostic performance. Results: all sonographic parameters were significantly different between the study groups, except for TD/(LD+APD). Optimal cut-off values of APD and LD/APD showed the best sensitivity and specificity. APD diameter equal or superior to 39.5 mm (95% CI, 36.2–42.8) had sensitivity of 0.70 (95% CI, 0.57–0.80), specificity of 0.71 (95% CI, 0.59–0.82) and accuracy of 0.75 (95% CI, 0.66–0.84). LD/APD equal or inferior to 2.05 (95% CI, 1.96–2.13) showed sensitivity and specificity of 0.70 (95% CI, 0.57–0.80) each and accuracy of 0.72 (95% CI, 0.62–0.81). Conclusions: several biometric uterine parameters at TVUS in fertile-aged women were statistically different between adenomyosis and non-adenomyosis uteri, though their optimal cut-off values showed low accuracy in diagnosing adenomyosis.

## 1. Introduction

Adenomyosis is a benign gynecological disease described by the presence of endometrial glands and stroma within the myometrium, as well as reactive hyperplasia and hypertrophy of the muscular layer [1].

Adenomyosis can be focal or more frequently diffuse and may involve junctional zone and/or outer myometrium [2,3,4]. When symptomatic, it can be the cause of abnormal uterine bleeding, pelvic pain and subfertility [2,3,4,5].

The reported prevalence of adenomyosis at pathological evaluation varies between studies from 21% to 36% according to different patients’ inclusion (e.g., age, symptoms, co-existent endometriosis) and histological criteria adopted [6,7,8]. However, because only a small, selected group of women undergo hysterectomy, the real prevalence of adenomyosis is likely underestimated, particularly in fertile-age women who have not accomplished childbearing and/or in patients with less advanced disease [9].

Therefore, non-invasive diagnosis of adenomyosis is clinically needed to also manage patients with actual or future desire of pregnancy. Transvaginal ultrasound (TVUS) should be considered the primary imaging tool in experienced hands, since it is an accurate, low cost and easily accessible method [10,11,12].

The aim to provide a standardized terminology for describing ultrasound images of normal and pathological myometrium, minimizing inter-operator variability, has been realized with the Morphological Uterus Sonographic Assessment (MUSA) consensus [2], which provided a list of sonographic features associated with adenomyosis [3]. Noteworthy, the importance of each item or their combinations for the TVUS diagnosis of adenomyosis has yet to be clearly defined. Among sonographic parameters, globular uterus is a qualitative assessment defined as a uterine configuration characterized by a rounded fundus and a more spherical shape [13,14]. Available studies evaluating its performance for adenomyosis diagnosis showed controversial results [10,11,15,16,17,18].

To the best of our knowledge, there are poor data and controversial results on diagnostic performance of some quantitative biometrical parameters that can differentiate adenomyosis uteri from non-adenomyosis ones [19,20].

The aim of this study is to compare uterine biometric parameters at TVUS between uteri with diffuse adenomyosis and uteri without adenomyosis and evaluate their potential role for the diagnosis of diffuse adenomyosis.

## 2. Materials and Methods

### 2.1. Study Protocol and Selection Criteria

The study followed an *a priori* defined study protocol and was designed as an observational, prospective, case-control study. The whole study was reported following the STROBE guidelines and checklist [21].

We enrolled all consecutive patients referring to our tertiary outpatient clinic for gynecological ultrasound between the 1 February 2022 and the 30 April 2022. We *a priori* defined as exclusion criteria: age less than 18 years, virgo status, postmenopausal status, ongoing or recent (less than 6 months) pregnancy, suspicion of gynecological malignancy, uterine malformations, previous surgery for adenomyosis or fibroids, focal adenomyosis lesions larger than 1 cm (focal adenomyosis/adenomyoma), and myomas larger than 1 cm.

### 2.2. Case and Control Goups

Cases group consisted of eligible women with sonographic criteria suggestive of adenomyosis according to the MUSA consensus [2,3] and presence of question mark sign [6].

Controls group was selected through a 1:1 ratio matching with cases for potential confounding factors (i.e., age, parity and hormonal therapy) and included consecutive eligible patients with no or one sonographic criteria suggestive of adenomyosis at routine evaluation in our Hospital during the study period.

The two study groups (adenomyosis uteri versus non adenomyosis uteri) were compared in terms of baseline and sonographic variables.

### 2.3. Study Outcomes

Primary outcomes were the comparisons of sonographic biometric parameters (absolute uterine diameters and simple and complex diameter ratios) between the two study groups.

Secondary outcomes included the diagnostic performance of optimal cut-off values of the investigated biometric parameters in diagnosing diffuse adenomyosis.

### 2.4. Patient Assessment and Ultrasound Details

Women received an accurate anamnestic evaluation, gynecological bimanual palpation, and 2D-3D transvaginal ultrasound scan. 

Baseline characteristics [e.g., age, body mass index (BMI), parity, hormonal therapy ≥3 months before study, previous surgery for endometriosis, moderate-to-severe pain symptoms defined as numerical rating scale equal or superior to 5, and heavy menstrual bleeding referred to as pictorial blood loss analysis chart ≥ 100) were obtained for all patients.

All TVUS examinations were conducted by highly trained expert sonographers using a 2-, 3-dimensional micro-convex endocavity transducer with a frequency range of 4 to 9 MHz (Voluson E8; GE Healthcare, Zipf, Austria). Ultrasound examinations were performed in order to detect the presence of any sonographic features suggestive of adenomyosis according to the MUSA consensus [2,3] and of question mark sign [6]. Sonographic features suggestive of adenomyosis according to the MUSA consensus include the presence of globular uterus appearance, asymmetrical thickening, hypoechogenic myometrial cysts, hyperechoic islands, fan-shaped shadowing, echogenic subendometrial lines and buds, translesional vascularity, irregular or interrupted junctional zone at 2D and 3D evaluations [18,22,23]. Adenomyosis was diagnosed when two or more of the above-mentioned sonographic criteria were present [15,24].

**Figure 1:**
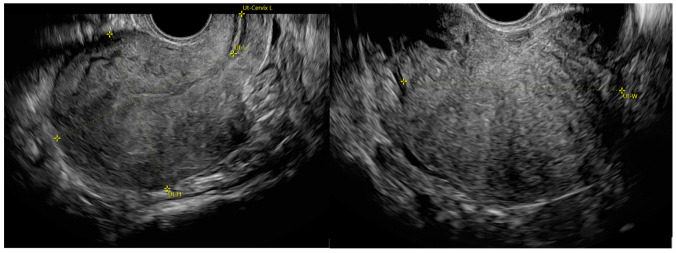
Standardized uterine measurement according to the MUSA consensus [2]. Longitudinal diameter (LD) is the entire uterine length measured as the sum of uterine corpus (Ut-L) and cervix length (Ut-Cervix L). Anteroposterior diameter (APD) and transverse diameter (TD) are measured in the sagittal (Ut-H) and transverse plane (Ut-W), respectively.

Uterine longitudinal (LD), anteroposterior (APD) and transverse (TD) diameters and volume were acquired at TVUS for all patients, and diameter sums, and simple and complex diameter ratios were calculated. In particular, assessed diameter sums were APD+TD and LD+APD, simple diameter ratios were LD/APD, LD/TD, and APD/TD, and complex dimeter ratios were LD/(APD+TD) and TD/(LD+APD). LD was measured as the entire uterine length: the sum of the uterine corpus (from the fundal serosal surface to the internal os) and the cervix length. The longest anteroposterior diameter was measured in the sagittal plane and the longest transverse diameter in the transverse plane (Figure 1). Volume of the uterus was calculated with the following formula: uterine corpus × APD × TD × 0.52.

### 2.5. Statistical Analysis

Categorical variables were summarized as frequencies and percentages, while numerical variables were summarized as mean ± standard deviation (SD) and median [interquartile range]. Baseline characteristics in the two study groups were compared with the Chi-squared test, Fisher’s exact test and t-test or Wilcoxon rank-sum (Mann–Whitney) test, where appropriate.

In order to investigate the presence of systematic differences in the biometric measurements of the uterus between the two study groups we performed the Wilcoxon rank-sum (Mann–Whitney) test. The measures that exhibited a significant association with the diagnosis of adenomyosis at Mann–Whitney test were further evaluated with nonparametric receiver operating characteristic (ROC) analysis. More specifically, the optimal cutoff value was determined using the Liu method, which maximizes the product of the sensitivity and specificity. 

Diagnostic accuracy was *a priori* categorized as null for AUC ≤ 0.5, low for 0.5 < AUC ≤ 0.75, moderate for 0.75 < AUC ≤ 0.9, high for 0.9 < AUC < 0.97, and very high for AUC ≥ 0.97, as previously reported [25].

All analyses were carried out using Stata software, version 17 (StataCorp, 2021, Stata Statistical Software: Release 17, College Station, TX, USA: StataCorp LP). All tests were two-sided, and the significance level was set at 0.05.

### 2.6. Ethical Statement and Informed Consent

The study protocol received approval by the local Ethics Committee (114/2022/Oss/AOUBo). All patients signed an informed consent before entering the study, and all data were anonymized.

## 3. Results

During the study period, we enrolled 56 women with sonographic diagnosis of diffuse adenomyosis as cases, and 56 matched patients without sonographic diagnosis of diffuse adenomyosis as controls.

Patients’ characteristics are presented in Table 1. The mean (±SD) age and BMI (±SD) of the entire study population were 37.6 (±7.7) and 24.0 (±3.3), respectively. In this case, 66 patients (59%) assumed hormonal therapy before entering the study. Most of the patients (68%) were nulliparous.

As reported in Table 2, all sonographic features suggestive of adenomyosis were significantly different between normal and adenomyosis uteri.

**Figure 2:**
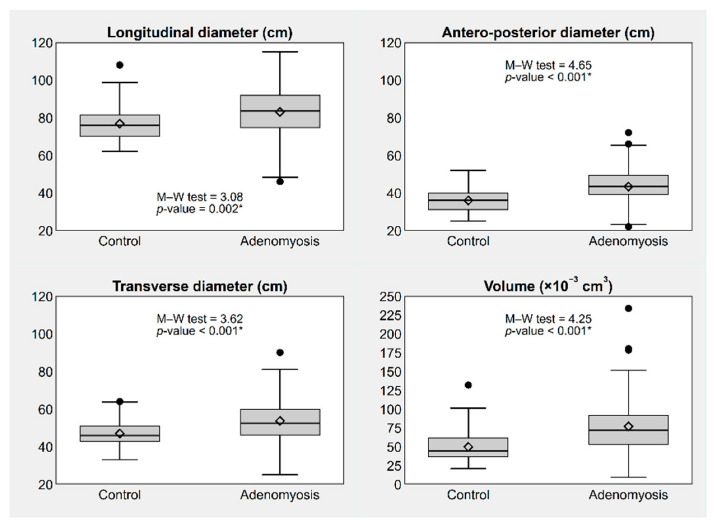
Box plots showing absolute uterine diameters and volume of the uterus, by presence or absence of adenomyosis. * *p*-value ≤ 0.05. Notes: The box represents the interquartile range (IQR), i.e., the distance between the 1st and the 3rd quartile (Q1, Q3); the bar and diamond inside the box represent the median and the mean, respectively; the whiskers represent the lower and upper adjacent values, i.e., the lowest observation ≥ Q1 − 1.5 × IQR and the largest observation ≤ Q3 + 1.5 × IQR; the circles represent the individual values observed outside the whiskers (outliers).

All uterine diameters and uterine volume were significantly different between the two groups (Figure 2).

**Figure 3:**
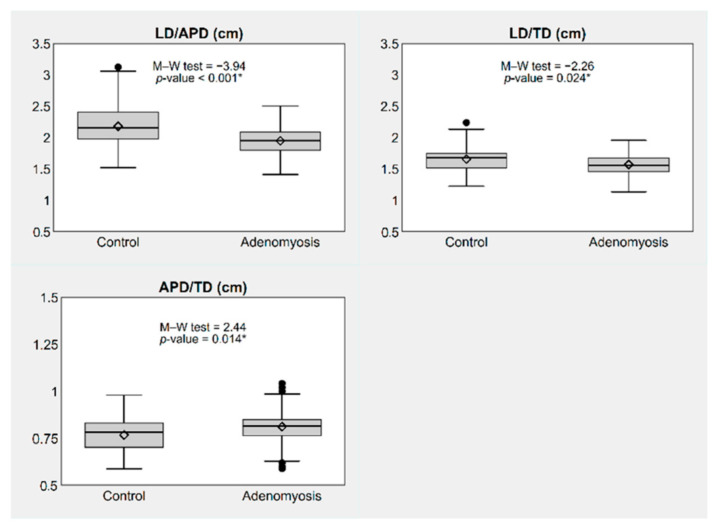
Box plots showing simple diameter ratios of the uterus, by presence or absence of adenomyosis. * *p*-value ≤ 0.05. Notes: The box represents the interquartile range (IQR), i.e., the distance between the 1st and the 3rd quartile (Q1, Q3); the bar and diamond inside the box represent the median and the mean, respectively; the whiskers represent the lower and upper adjacent values, i.e., the lowest observation ≥ Q1 − 1.5 × IQR and the largest observation ≤ Q3 + 1.5 × IQR; the circles represent the individual values observed outside the whiskers (outliers). Abbreviations: LD, longitudinal diameter; APD, antero-posterior diameter; TD, transverse diameter.

**Figure 4:**
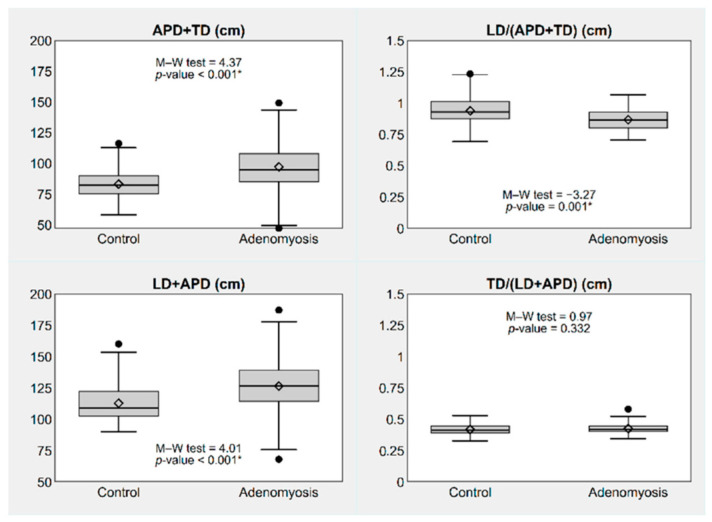
Box plots showing diameter sum and complex diameter ratios of the uterus, by presence or absence of adenomyosis. * *p*-value ≤0.05. Notes: The box represents the interquartile range (IQR), i.e., the distance between the 1st and the 3rd quartile (Q1, Q3); the bar and diamond inside the box represent the median and the mean, respectively; the whiskers represent the lower and upper adjacent values, i.e., the lowest observation ≥ Q1 − 1.5 × IQR and the largest observation ≤ Q3 + 1.5 × IQR; the circles represent the individual values observed outside the whiskers (outliers). Abbreviations: LD, longitudinal diameter; APD, antero-posterior diameter; TD, transverse diameter.

Similarly, simple and complex diameter ratios were statistically different between the two groups, except for TD/(LD+APD) (Figure 3 and Figure 4).

As illustrated in Table 3, optimal cutoff of each biometric parameter demonstrated a low discriminative value for the diagnosis of adenomyosis.

Among all investigated parameters, the optimal cut-off values of APD and ratio LD/APD showed the best sensitivity and specificity. In particular, APD diameter equal or superior to 39.5 mm (95% CI, 36.2–42.8) had sensitivity of 0.70 (95% CI, 0.57–0.80), specificity of 0.71 (95% CI, 0.59–0.82) and accuracy of 0.75 (95% CI, 0.66–0.84). LD/APD ratio equal or inferior to 2.05 (95% CI, 1.96–2.13) showed sensitivity and specificity of 0.70 (95% CI, 0.57–0.80) each and accuracy of 0.72 (95% CI, 0.62–0.81).

## 4. Discussion

This study demonstrated that quite all biometric parameters [except for TD/(LD+APD)] at TVUS were statistically different between adenomyosis and non-adenomyosis uteri of fertile-aged women attending to tertiary referral outpatient center. Nevertheless, optimal cut-off values of these biometric parameters showed a low accuracy in diagnosing adenomyosis. Among biometric parameters, APD diameter equal or superior to 39.5 mm and LD/APD ratio equal or inferior to 2.05 showed the best combination of sensitivity and specificity.

Nowadays, TVUS performed by expert operators can be considered the first-line imaging technique to diagnose adenomyosis [10,11,12]. In order to standardize TVUS for diagnosing adenomyosis, MUSA consensus was proposed to detect any direct and indirect ultrasonographic features of the disease [2,26]. Myometrial cysts, hyperechogenic islands and echogenic subendometrial lines and buds are direct sonographic manifestations of the presence of ectopic endometrium in the myometrium; while indirect features, such as globular uterus, asymmetrical myometrial thickening, fan shaped shadowing, translesional vascularization and irregularity of the junctional zone, reflect myometrial hyperplasia or hypertrophy consequent to ectopic endometrial tissue implantation into the myometrium, diffuse increased myometrial vascularity and invagination of basalis endometrium in the junctional zone [27].

Among indirect feature, globular uterus showed poor pooled sensibility, moderate pooled specificity and a low accuracy in diagnosing adenomyosis using TVUS [10,11,12]. However, a lack of a globally accepted ultrasound description and objective biometrical characterization of the globular uterus could have contributed to its low diagnostic performance and inter-operator agreement [14,28]. In order to make a shift from a qualitative sign to a more reproducible and objective quantitative diagnostic parameter for diffuse adenomyosis, we sought to find an accurate and simple quantitative biometric uterine parameter.

According to a recent Delphi procedure, globular shape of the uterus was not defined as a merely enlargement of the uterus in size, but as a divergence of myometrial serosa of the anterior and/or posterior wall from the cervix instead of following a trajectory parallel to the endometrium [26]. In agreement with this definition, despite we observed an increase of all three uterine diameters and organ volume, APD showed a higher increase rather than other diameters. This observation suggests the adenomyosis uteri tend to enlarge mainly in an anteroposterior direction rather than in longitudinal or transversal one.

Before our study, Mooney et al. investigated the diagnostic accuracy of the ratio between antero-posterior diameters of uterus and cervix, named myometrial-cervical ratio, in a series of pre- and post-menopausal women undergone hysterectomy for benign non obstetric conditions [19]. Despite promising results in their first retrospective study, validation prospective study on women requiring hysterectomy revealed low accuracy, sensitivity and specificity, in particular when fibroids were not excluded in the calculation of the uterine AP diameter [20].

Similarly, in our study on pre-menopausal women without diagnosis of uterine fibroid over 1 cm, despite several uterine biometric parameters and their ratios showed significant differences between adenomyosis and non-adenomyosis uteri, they presented a low diagnostic performance for diagnosing diffuse adenomyosis at TVUS. These findings may be related to high heterogeneity of the disease, which can occur with multiple sonographic and/or clinical patterns [14,27,29]. 

Despite prospective design and novelty of this study, it has some limitations. First, we decided to have as reference standard sonographic diagnosis by expert operators according to the presence of at least two sonographic criteria of diffuse adenomyosis, as recommended by the MUSA consensus [2,26]. Although pathological examination of the uterus is considered the gold standard, [30,31] hysterectomy is only possible in a small percentage of patients potentially affected by adenomyosis (i.e., symptomatic women with no desire of pregnancy or post-menopausal patients); moreover, no consensus about histological criteria exists. However, post-histological diagnostic era of adenomyosis has come and non-invasive methods should be clinically used to diagnose and manage fertile-age patients using a tailored approach on a case-by-case basis [4,29,32,33,34]. Second, the monocentric design at a tertiary referral center for endometriosis and chronic pelvic pain potentially limits the generalization of our findings. However, we selected uterine biometric parameters according to the MUSA consensus which are easily reproducible. Third, small sample size may limit the reliability of our results. Nevertheless, we adopted strict inclusion/exclusion criteria for avoiding confounding factors on uterine diameters.

Further larger studies are needed to confirm our data and investigate the role of sonographic biometric parameters of the uterus in diagnosing of adenomyosis in different settings and conditions.

## 5. Conclusions

Although several biometric uterine parameters at TVUS in fertile-aged women were statistically different between adenomyosis and non-adenomyosis uteri, their optimal cutoff values showed low accuracy in diagnosing adenomyosis, potentially limiting their application in the clinical practice.

Future larger studies are needed to further evaluate their usefulness as diagnostic markers of diffuse adenomyosis.

## Figures and Tables

**Table 1:** Baseline characteristics of the entire study population and of the two study groups.

Characteristic	All (*n* = 112)	Adenomyosis		
		Yes	No	*p*-Value
		(*n* = 56)	(*n* = 56)	
Age, y	37.6 ± 7.7	37.5 ± 7.7	37.7 ± 7.7	0.913
BMI	24.0 ± 3.3	23.6 ± 3.3	24.2 ± 3.4	0.098
Age group, y				0.996
≤25	9 (8%)	5 (9%)	4 (7%)	
26–30	15 (13%)	7 (13%)	8 (14%)	
31–35	12 (11%)	6 (11%)	6 (11%)	
36–40	34 (30%)	16 (29%)	18 (32%)	
41–45	24 (21%)	13 (23%)	11 (20%)	
>45	18 (16%)	9 (16%)	9 (16%)	
Parity				0.555
0	76 (68%)	38 (68%)	38 (68%)	
1	25 (22%)	11 (20%)	14 (25%)	
2	11 (10%)	7 (12%)	4 (7%)	
Hormonal therapy for ≥3 months before study				0.157
None	46 (41%)	23 (41%)	23 (41%)	
Progestin	34 (30%)	21 (38%)	13 (23%)	
Intrauterine device	17 (15%)	8 (14%)	9 (16%)	
Estrogen/progestin	15 (13%)	4 (7%)	11 (20%)	
Previous surgery for endometriosis	42 (38%)	30 (54%)	12 (21%)	<0.001 ^a^
Pain symptoms (NRS ≥ 5)				
Dysmenorrhea	14 (13%)	9 (16%)	5 (9%)	0.253
Dyspareunia	15 (13%)	11 (20%)	4 (7%)	0.052
Ovulation pain	4 (4%)	3 (5%)	1 (2%)	0.618
Chronic pelvic pain	7 (6%)	6 (11%)	1 (2%)	0.113
Dysuria	1 (1%)	0 (0%)	1 (2%)	1.000
Dyschezia	3 (3%)	3 (5%)	0 (0%)	0.243
Heavy menstrual bleeding (PBAC ≥ 100)	9 (8%)	8 (14%)	1 (2%)	0.016 ^a^

^a^ *p*-value ≤ 0.05. All characteristics were expressed as number (percentage). Abbreviations: y, years; NRS, numerical rating scale; BMI: body mass index; PBAC: Pictorial Blood Assessment Chart.

**Table 2:** Ultrasonographic characteristics of the entire study population and of the two study groups.

Characteristic	All (*n* = 112)	Adenomyosis		
		Yes	No	*p*-Value
		(*n* = 56)	(*n* = 56)	
Globular uterus	46 (41%)	41 (73%)	5 (9%)	<0.001 ^a^
Fan-shaped shadows	38 (34%)	37 (66%)	1 (2%)	<0.001 ^a^
Hyperechogenic islands	36 (32%)	36 (64%)	0 (0%)	<0.001 ^a^
JZ interruption/irregularities	36 (32%)	30 (54%)	6 (11%)	<0.001 ^a^
Anechoic myometrial cysts	35 (31%)	34 (61%)	1 (2%)	<0.001 ^a^
Trans-lesional vascularity	33 (29%)	33 (59%)	0 (0%)	<0.001 ^a^
Echogenic sub-endometrial lines and buds	32 (29%)	32 (57%)	0 (0%)	<0.001 ^a^
Asymmetry of the uterine walls	52 (46%)	45 (80%)	7 (13%)	<0.001 ^a^
QM sign	18 (16%)	18 (32%)	0 (0%)	<0.001 ^a^

^a^ *p*-value ≤0.05. Notes: Values are *n* (%), or mean ± standard deviation and median [interquartile range]. Abbreviations: JZ: junctional zone; QM: question mark.

**Table 3:** Results of nonparametric receiver operating characteristic (ROC) analysis conducted on the absolute uterine diameters, simple and complex diameter ratios and volume of the uterus to discriminate adenomyosis.

Measurement	Optimal Cutoff	Sensitivity at Cutoff	Specificity at Cutoff	AUC
	Est. (95% CI)	Est. (95% CI)	Est. (95% CI)	Est. (95% CI)
LD, cm	78.5 (73.6, 83.4)	0.64 (0.51, 0.76)	0.66 (0.53, 0.77)	0.67 (0.57, 0.77)
APD, cm	39.5 (36.2, 42.8)	0.70 (0.57, 0.80)	0.71 (0.59, 0.82)	0.75 (0.66, 0.84)
TD, cm	52.5 (46.6, 58.4)	0.50 (0.37, 0.63)	0.82 (0.70, 0.90)	0.70 (0.60, 0.80)
Volume, ×10^3^ cm^3^	71.1 (45.6, 96.6)	0.79 (0.66, 0.87)	0.64 (0.51, 0.76)	0.73 (0.64, 0.83)
LD/APD, cm	2.05 (1.96, 2.13)	0.70 (0.57, 0.80)	0.70 (0.57, 0.80)	0.72 (0.62, 0.81)
LD/TD, cm	1.67 (1.56, 1.78)	0.73 (0.60, 0.83)	0.52 (0.39, 0.64)	0.62 (0.52, 0.73)
APD/TD, cm	0.76 (0.70, 0.81)	0.77 (0.64, 0.86)	0.48 (0.36, 0.61)	0.63 (0.53, 0.74)
APD+TD, cm	90.5 (84.4, 96.6)	0.66 (0.53, 0.77)	0.77 (0.64, 0.86)	0.74 (0.65, 0.83)
LD/(APD+TD), cm	0.90 (0.85, 0.96)	0.64 (0.51, 0.76)	0.64 (0.51, 0.76)	0.68 (0.58, 0.78)
LD+APD, cm	117.5 (107.8, 127.2)	0.70 (0.57, 0.80)	0.68 (0.55, 0.79)	0.72 (0.62, 0.82)

Abbreviations: Est., estimated; CI, confidence interval; AUC, area under the ROC curve; LD, longitudinal diameter; APD, anteroposterior diameter; TD, transverse diameter.

## Data Availability

The data presented in this study are available on request from the corresponding author. The data are not publicly available due to the need of privacy maintenance of patients.
